# American Dental Association

**Published:** 1885-11

**Authors:** 


### AMERICAN DENTAL ASSOCIATION.

TWENTY-FIFTH ANNUAL MEETING, HELD AT MINNEAPOLIS, MINN., AUGUST 4, 5, 6, AND 7, 1885.

REPORTED FOR THE INDEPENDENT PRACTITIONER, BY "MRS. M. W. J."

(Continued from page 538.)

### WEDNESDAY AUGUST 5TH—EVENING SESSION.

Section V—Anatomy, Histology, and Microscopy—was called. Dr. Frank Abbott, of New York, read a paper entitled "Studies of the Pathology of Enamel of the Human Teeth, with special reference to the Etiology of Caries."

The essayist first described the micro-stereopticon of Stricker, of Vienna, an instrument by which the field of an ordinary slide, magnified 12,000 diameters, is projected upon a screen so that a thousand persons can study it at once. Theories regarding tooth-structure are thus proved or disproved. Dr. Abbott said that he expected to receive one of these instruments in a few weeks, for the further prosecution of his researches in this direction. He also said that he was indebted to his friend and teacher, Carl Heitzman, for the very fine micro-photographic illustrations of his present paper, by which he was able to prove the truth of the positions taken.

He spoke of the very great importance of the study of the Pathology and Etiology of Caries. That there was a marked difference, not only in individuals, but also in races, in the liability of teeth to decay. That civilization had been accused of being a prominent factor of causation, but that this would not explain the rapidly growing tendency towards this disease under all circumstances. That there must be an anatomical sub-stratum, aside from acquired or local causes, the latter being mere auxiliary agents fostering more remote causes. He had examined a large number of specimens, ground into thin slabs, with the soft parts, their living matter, carefully preserved, the results being highly satisfactory in explaining the tendency to decay as due to deficiency in anatomical structure. Before entering into the description of anomalies, he described the structure of normal enamel and its relations to dentine, as discovered by Bodecker, the connection between the dentinal and enamel fibres, the distribution of living matter in tooth-substance, even in the interstices separating the minute subdivisions of the enamel rods, thus raising the enamel to the dignity of living tissue, instead of a mere deposit of calcareous elements. He said the dentinal canaliculi upon entering the enamel often became enlarged in pear-shaped cavities, containing protoplasm, which was in direct connection with the termination of the dentinal fibres, and on the periphery with the fibres of living matter of the enamel, these enlargements being more regular in the teeth of young persons.

The first plate represented a temporary molar, with a protrusion of dentine into the enamel, with a fluted surface, the centre of the protrusion occupied by protoplasmic formation, the enamel nearest the protrusion destitute of prisms, the zone above the protrusion but slightly brownish, while the prisms of the enamel exhibited a very distinct brown pigmentation.

The second plate represented a permanent canine with a pear-shaped or conical protuberance without distinct boundary on the buccal surface, occupying nearly one-half the enamel, the adjacent enamel showing indistinct rods, but occupied by brownish, globular fields, resembling the interglobular spaces of dentine. The rods were slightly pigmented, and more wavy than normal. There were no interglobular spaces in the dentine of this tooth.

The third plate was a diagram showing the stratification of enamel layers varying in width, the broadest portion always corresponding to the cusps, the narrowest to the neck of the tooth, the enamel rods of the cusp-layers invariably vertical to the surface, while those of the neck-layers may be parallel to the surface, this construction sometimes varying at the outer periphery of the enamel. Higher powers of the microscope show that the lines of stratification do not alter the general course of the enamel prisms, an exception occurring only at the peripheral portions of enamel occupied by the tapering ends of the neck-layers, which may exhibit enamel rods almost parallel with the surface of the enamel, while at the outer periphery of the cusp-layers the enamel rods would invariably be more or less vertical.

The stratification of the enamel is of the utmost importance for the understanding of pigmentation and granulation, these both corresponding with the general strata of the enamel, showing in a longitudinal section a fan-like appearance. The stratification of this tissue bears close relation to the history of its development. The first enamel cap of temporary teeth is broadest in the direction of the cusp, and tapering towards the future neck, subsequent layers forming on the plan of the first.

Illness of the mother, before delivery, or of the infant after delivery, might cause an interruption in its construction, leading to stratification, pigmentation, and granulation, these conditions invariably involving a deficient deposition of lime-salts.

Figure four illustrated the irregular course of pigmented enamel rods. In normal enamel, longitudinal sections exhibit slightly wavy rods, interlaced by small bundles in a transverse direction, the curvature becoming gradually less, till it is nearly straight, close to the surface. Where there are deviations from the rule, they lose all regularity, oblique and longitudinal blending, as in the graining of lignum-vitæ. In such cases the curvature of the rods may remain marked up to the surface, showing groups of transverse sections at the outer surface, perhaps on one portion only, the rest of the enamel being normal. With this curly appearance pigmentation is combined, and the interstices between the rods are wider than normal, all indicating deficient calcification. The dentine is also freely supplied with interglobular spaces, also another sign of deficient calcification. The friability of such enamel is so great that it is extremely difficult to obtain an unbroken slab. With low powers of the microscope the broken ends of the enamel rods look as if corroded. The enamel rods are unusually narrow, the interstices correspondingly wide, and their tenants—the enamel fibres—very prominent. The reticulum near the inter-zonal layer is also very prominent. These conditions may occur both in temporary and in permanent teeth, and may be combined with pigmentation.

Another result of deficient calcification is seen in the congenital white or yellow spots, which, if broken into, are found of the consistency of chalk. This is miscalled "white decay," but is a congenital defect due to deficient calcification, and is closely allied to the rows of pits, with no enamel in the bottom of the depressions. These are also congenital and due to the same cause. Some portions, or all of the crown, may be covered by a brown or yellow substance, which is not enamel, but which is soft and easily removed, leaving the dentine exposed and extremely sensitive. These might all be termed exaggerated cases of pigmentation. The form which is most common presents to the naked eye only a slight yellow-brown discoloration. The microscope shows that the pigmentation occurred during the formation of the structure. If in non-stratified enamel, there is no demarkation of the brown spots, but faintly marked offshoots taper towards the dentine. In stratified enamel the deepest stain corresponds to the boundary of the strata, tapering towards the neck, fading towards the proximal line, showing a fan-like configuration in longitudinal sections. This pigmentation may invade all the layers of enamel, being perhaps more marked in the deepest portions. Above, the microscope shows that the brown discoloration concerns the basis-substance of the enamel rods only; that the widened interstices are due to deficiency in formation, and not merely to contrast in color; that the transverse lines are also enlarged; that the enamel fibres are more conspicuous than even in normal enamel of temporary teeth. Pigmented enamel also readily stains with an ammoniacal solution of carmine. It is not yet known what disturbance of the enamel organ, interfering with the proper construction of the basis-substance and the deposition of lime-salts, causes the brown discoloration. All pigments of the body depend upon the coloring matter of the blood, but careful studies in the history of the development of enamel must be made, before the solution of this question can be reached. One point is positive, viz., that pigmentation is congenital, invading both permanent and temporary teeth, acquired pigmentation being rare except as the result of caries. Caries invading spots congenitally pigmented causes an orange discoloration, which readily takes the carmine stain, the congenital brown, acquired orange, and artificial red mingling in beautiful shadings.

The granulation of enamel is a peculiar pathological condition by no means rare, and consists of pear, spindle and club-shaped spaces in the middle of the substance of the enamel, as well as at the junction of the dentine and enamel, as heretofore recognized. These spaces are enlargements of the interstices between the prisms and the tenants of the interstices. Plate VI represented this condition under high powers of the microscope.

The interprismatic spaces of the enamel bear some resemblance to the interglobular spaces of the dentine, both conditions being present in highly marked degree in one specimen. The deficiency of basis-substance and lessened amount of lime-salts render granular enamel both brittle and prone to decay. Stratification, pigmentation and granulation, are all pathological conditions of the enamel, meaning a deficient formation of the basis-substance and also decreased deposition of lime-salts, and are of the highest importance in the etiology of caries.

The conclusion reached was, that ailments of either the mother during gestation, or of the infant during the earliest periods of life, cause these anomalies; that they are more common in high life and in persons of refined habits, debilitated by the luxuries of civilization, than among hard-working people. The pathology of enamel has no literature.

DISCUSSION.

*Dr. C. N. Pierce*—Said that he wished to congratulate the association on the opportunity they had enjoyed, through this paper, of examining diagrams illustrating vital conditions, such as were not often offered to any society. He desired to call attention to the fact that number One illustrated conditions that must have been confirmed in utero. Between the dentine and enamel the line of first calcification must have taken its shape as early as the fifteenth or sixteenth week in embryo life. It must have had its peculiar form at that time before being rendered permanent by the deposition of salts of lime. This afforded a valuable lesson in the vitality of structure, and how readily it is modified by lack of nutrition. The abnormal projection of dentine-structure was doubtless due to some peculiarity of nutrition currents at that early period. It teaches the readiness with which tooth-structure is modified by systemic conditions.

Diagram No. 3 afforded the same index as the concentric lines of the tree-periods of growth. In the enamel of the diagram referred to there were clearly indicated periods of growth and arrest—nutrition and cessation—affording another proof of the liability of the teeth to be influenced by systemic conditions. And so on through all the diagrams, each representing a special phase, but all teaching the same lesson—the undoubted modification of these hard structures by systemic conditions.

*Dr. A. H. Thompson*—Desired to ask Dr. Abbott to say something further about the white spots on enamel.

*Dr. Abbott*—The only evidence we have of the diminution of lime-salts is the greater amount of organic material; it must be that much short of inorganic elements. The lime-salts are loose, can be scraped off with the thumb nail, will almost break off by looking at it. The yellow spots look like enamel, but are not; the interprismatic spaces are two or three times larger than normal; the lime-salts then must be less; the relative proportion is changed. There is a certain amount of satisfaction in having a knowledge of disease, even if we cannot cure. With reference to "sterilized earth," what I said was a quotation from the French, and not cited as authority.

*Dr. T. W. Brophy*—Dr. Abbott said that all defects previous to eruption were congenital. According to some observers, as Prof. Eames, of St. Louis, endorsed by Prof. Black, some defects are the result of absorption, similar to that occurring in the roots of deciduous teeth, and due to the same agent as that which absorbs the roots. Such an absorption may take place; I have seen the destruction of the enamel of incisors from this cause.

*Dr. Abbott*—The condition I referred to is, in my judgment, impossible after eruption—the very dark, porous, granular condition of a line running out upon the surface. It requires no stretch of imagination to see that something might flow in and affect all the tissues along that line. It may be possible to show that it is a case of absorption, but I think it stretching a point; I cannot see how it could occur similarly to the absorption of the root.

*Dr. Keith*—Said that he had had a peculiar case in practice. He had a regulating plate in the mouth of a little girl, which covered one of the deciduous teeth, the one to be replaced by the first bicuspid. She was absent two or three weeks. When he removed the plate he found the crown of the deciduous tooth imbedded in the vulcanite plate, with every particle of the dentine dissolved out; it seemed as if cells had grown up above the margin of the gum, and eaten out the dentine, without touching the thin margin of enamel.

*Dr. Atkinson*—The dentine must be reduced to fluidity before it can be taken up by absorbents. You say that the carneous body does the work! No more than the liver secretes liver. Dr. Abbott gave me an adult tooth that had lost two-thirds of the length of the root; it was perforated as if by drill holes. He had not had time to cut it yet, but intended to do so. It was a case of retrogressive metamorphosis, but we cannot understand that.

*Dr. L. C. Ingersoll*—Read a paper entitled "The Alveolar Dental Membrane; Unity or Duality, Which?"

Handing his specimens to be passed around, he said that he had no doubt of his ability to read his paper while the specimens were being examined, but he was not so certain of being heard or understood.

The following is a summary of his argument: He said that the dental membrane offered a subject which commended itself to our consideration; that upon the condition of this membrane depended the health, safety, and comfort of the teeth. Unless it was in a healthy condition the pulp might be capped and the teeth filled in vain; but if the membrane was vigorous the crown might decay, and the pulp die, and yet a serviceable tooth be built upon the root. If the membrane fails the whole structure is gone. Much study is given to alveolar abscess, to pyorrhœa, but the histology of the membrane as a dental tissue is scarcely referred to. The enamel, the dentine, the cementum, the pulp—three hard and one soft tissue springing from the dental follicle—are each fully treated of, but the membrane has but passing notice. It is a subject which is full of difficulties. Does it appertain to the dental follicle? Early anatomists regarded the tooth as bone, and the alveolar periosteum as an osseous membrane. In the development of the tooth the crown is first formed, then the neck, then the root. The follicular neck is a point of particular interest. He quoted from Legros, Magitot, Tomes and other authors, on this point.

The alveolar walls develop simultaneously with the root. Proceeding out from the base of the follicle is a line of cells, the formative point of cementum, and another line, the formative organ of the alveolar walls. One develops down to form the socket, the other forms the cementum, the two layers being contiguous. The question before us is—is the tissue known indifferently as dental periosteum or as alveolar membrane, single or dual? The earliest writers say nothing on the subject. Later authors dismiss it with a few words, speaking of it as a connective tissue well-supplied with blood vessels investing the root and lining the socket. Different names are given to it, showing varying opinions, but it cannot be studied from authors, they are so shy of mentioning it. Where a mucous membrane rests upon bone, it is intimately connected with it. Analogy would lead us to expect that a true alveolo dental membrane would be in two layers; demonstration proves it. When a tooth is extracted the root is covered with a membrane, and the socket is lined with membrane. If the membrane exists in two distinct layers, have they the same origin?

The alveolar lining must be referred to the osseous system. The organ which forms the cementum cannot be the same as that which forms the alveolar walls. They are intimately united, but not one; they are distinct and separable; two layers of distinct origin and performing different offices; an outer layer of connective tissue, with blood vessels, an inner layer of a fine network of cells, there being a marked difference in their histological character, the fibrous elements of the outer passing insensibly into the network of the inner—the peridentium of the tooth, the periosteum of the bone. However close in juxtaposition, they are widely apart in function. Tomes says that two membranes may be assumed, but would be extremely difficult to demonstrate; that the fibrous elements of the outer portion blend so insensibly into the bands of network of the inner part, that neither sight nor touch, nor any other sense can show any distinction, leaving only a mere inference that it is two rather than one.

Dr. Ingersoll said that while investigating this subject he visited Dr. Black, to see if he had any specimens that would throw light on the subject. He found one specimen that was perfect in all its parts, and in which the osseous membrane and the cemental membrane appeared entirely distinct. First, there was a fine network in the meshes of the cemento-blasts, then bundles of fibres in flattened bands, side by side, passing in oblique and curved lines toward the bone. The reticulated osteo-blasts were much larger than the cemento-blasts. None of the straight lines of the peridental membranes were found in the alveolar membrane.

There are thus two membranes, with special functions, the outermost membrane forming the bone, the innermost the cementum. They are similar, but not the same. Tomes says the development of bone might, with some modification, be applied to the development of cementum. Have they different sources of mineral and vascular supply? If the membrane is dual, two sources would be expected; from the pulp on the one hand, from sub-mucous tissues on the other. Pathology will reveal the difference in ex-ostosis and ex-cementosis. Irritation follows the course of the blood-vessels. Under the influence of irritants the root membrane takes on abnormal action ; this is one cause of ex-ostosis—really ex-cementosis. If the nerves and blood-vessels follow the course of the membrane, and it is not dual, why are not both layers affected ? Why do we not have both true ex-ostosis or enlargement of the socket, and also ex-cementosis or enlargement of the root, and consequent tooth-anchylosis? When two roots are ex-ostosed, the two grow together by impingement, but never the socket and root; hence there is no osseous union. Portions of the socket are removed, and inflammation of the membrane induces hypertrophic conditions. Finally, he drew the following conclusions :

1st—The alveolar and dental membranes are not identical; the root membrane is in separate layers.

2d—The two layers have not the same origin ; they originate respectively in the osseous system, and in the dental follicle.

3d—They differ in structure ; banded and reticular.

4th—They have different functions ; one forming the socket, the other the root.

5th—They have different sources of vascular and mineral supplies.

6th—Pathology points to the same fact; there is no cemented union between the root and the socket.

7th—The rapid mineral absorption of the root, without exfoliation, is due to the special nature of the dental membrane, as distinct from the membrane of the socket.

Thus surgery, pathology, and physiology all point to the same conclusion—the duality of the alveolo-dental membrane.

On motion the society adjourned to Thursday at 9 A. M.

(TO BE CONTINUED.)

